# Adjuvant Treatment for Breast Cancer Patients Using Individualized Neoantigen Peptide Vaccination—A Retrospective Observation

**DOI:** 10.3390/vaccines10111882

**Published:** 2022-11-08

**Authors:** Henning Zelba, Alex McQueeney, Armin Rabsteyn, Oliver Bartsch, Christina Kyzirakos, Simone Kayser, Johannes Harter, Pauline Latzer, Dirk Hadaschik, Florian Battke, Andreas D. Hartkopf, Saskia Biskup

**Affiliations:** 1 Zentrum für Humangenetik Tübingen, 72076 Tuebingen, Germany; 2 CeGaT GmbH, 72076 Tuebingen, Germany; 3 Cecava GmbH, 72076 Tuebingen, Germany; 4 Department of Obstetrics and Gynaecology University of Tuebingen, 72074 Tuebingen, Germany

**Keywords:** vaccination, peptide, neoantigen, breast cancer

## Abstract

Breast cancer is a tumor entity that is one of the leading causes of mortality among women worldwide. Although numerous treatment options are available, current explorations of personalized vaccines have shown potential as promising new treatment options to prevent the recurrence of cancer. Here we present a small proof of concept study using a prophylactic peptide vaccination approach in four female breast cancer patients who achieved remission after standard treatment. The patients were initially analyzed for somatic tumor mutations and then treated with personalized neoantigen-derived peptide vaccines. These vaccines consisted of HLA class I and class II peptides and were administered intracutaneously followed by subcutaneous application of sargramostim and/or topical imiquimod as an immunological adjuvant. After an initial priming phase of four vaccinations within two weeks, patients received monthly boosting/maintenance vaccinations. Chemotherapy or checkpoint inhibition was not performed during vaccination. One patient received hormone therapy. The vaccines were well tolerated with no serious adverse events. All patients displayed vaccine-induced CD4+ and/or CD8+ T-cell responses against various neoantigens. Furthermore, all patients remained tumor-free and had persistent T-cell responses, even several months after the last vaccination, suggesting the potential of peptide vaccines as an immunosurveillance and long term prophylaxis option.

## 1. Introduction

Notable advances have been achieved in breast cancer prevention, diagnosis, and treatment in recent years [1,2,3,4]. Current treatment involves a selection or combination of surgery, chemotherapy, radiotherapy, hormone therapy, targeted therapy, and immunotherapy, designed to impede tumor growth, proliferation, and metastasis, and to promote tumor cell death [5]. These strategies are dependent on histopathological, biological, and genetic markers [6]. The classification of breast cancer is based on the expression of three distinct receptors on tumor cells: estrogen receptor (ER), progesterone receptor (PR), and human epidermal growth factor 2 receptor (HER2). These three receptor types play a key role in prognosis and treatment options.

Despite the substantial progress in the diagnosis and treatment of breast cancer, recurrence rates of around 20% cause concern [7]. A further complicating matter is the heterogenous nature of breast cancer displaying a complex diversity of tumors in patients, beyond ER/PR/HER-based classification [8,9]. Comprehensive genetic sequencing to characterize cancer cells is ultimately necessary to select effective treatment options [10,11,12,13].

Although breast carcinomas, in general, comprise an intermediate tumor mutational burden [14], some individual tumors show relatively high TMB values as well as a relevant infiltration of lymphocytes [15]. Cancer mutations can lead to the formation of tumor-specific neoantigens. These neoantigens are presented on HLA molecules as non-self-antigens, allowing the immune system to recognize and combat tumor cells. Thus, in addition to approved therapeutic options, it is also reasonable to target cancer neoantigens in breast carcinoma.

Because spontaneous anti-tumor immune responses are only infrequently observed [16], vaccination, with its potential to induce and/or reactivate anti-tumor immunity, has been increasingly applied in recent years [17]. Cancer vaccine design involves the selection of relevant target antigens and the application as well as the selection of an effective immunological adjuvant [18]. Neoantigens can be administered in different formats including peptides, recombinant viruses, DNA and RNA, or preloaded in vitro on dendritic cells [19,20]. We decided to use a peptide-based vaccine as they have shown encouraging progress in the stimulation of T-cells [21]. By mixing short (8–11 amino acid residues) and long (11–30 residues) peptides, we were aiming for presentation on both MHC class I and II molecules [11,22]. Hence, priming and/or reactivation of cytotoxic CD8+ T-cells, as well as CD4+ Helper T-cells, was targeted.

Here we present a retrospective analysis of personalized peptide vaccination in four breast cancer patients that were tumor-free after standard therapy. Neoantigen-derived peptide vaccines were designed to induce T-cell responses against individual tumor-specific antigens. The aim of this analysis was to show the feasibility, safety, and efficacy, in terms of immunogenicity, of a personalized peptide vaccine approach.

## 2. Materials and Methods

### 2.1. Patients

Four female tumor-free breast cancer patients volunteered to participate in an individual complementary adjuvant treatment attempt with personalized peptide vaccines at the *Zentrum fuer Humangenetik*, Tuebingen, Germany. At the time of the first vaccination, all patients had early or locally recurrent non-metastatic breast cancer and were successfully treated via surgery (R0). Clinical data were collected retrospectively from patients’ records. All patients gave written informed consent for treatment and retrospective analysis. This analysis was approved by the local ethics committee (Ethik-Kommission an der Medizinischen Fakultät der Eberhard-Karls-Universität und am Universitätsklinikum Tübingen; 434/2021BO2).

### 2.2. DNA Sequencing and Bioinformatic Analyses

Identification of individual neoantigens was performed as previously described [23]. DNA was extracted from formalin-fixated paraffin-embedded (FFPE; AllPrep DNA/RNA FFPE Kit; Qiagen, Hilden, Germany) tumor specimens and from EDTA blood (DNeasy Blood & Tissue Kit; Qiagen, Hilden, Germany). Sequencing libraries were prepared from each sample using the Agilent SureSelect workflow (Agilent, Santa Clara, CA, USA) and enriched with either Agilent SureSelect Exome 5 or 6, or a custom-design tumor panel covering 742 genes. Library preparation and capture were performed according to the manufacturer’s instructions and paired-end sequencing was performed on a HiSeq2500 or NovaSeq6000 (Illumina, San Diego, CA, USA).

Sequence variants were called with a minimum variant allele frequency of 5%. Resulting variants were annotated with population frequencies from public databases (dbSNP and EVS) and an internal database, with functional predictions from dbNSFP, with publications from HGMD^®^, and with transcript information from Ensembl, RefSeq, and CCDS. Blood and tumor data were analyzed comparatively to determine germline/somatic status for each variant.

Somatic variants were phased and the resulting coding sequences translated into the local amino acid sequence context to generate epitope candidates. The patient’s HLA class I type was identified using OptiType [24]. HLA-epitope binding affinity was predicted and selection of epitopes was performed using an in-house developed and proprietary neoepitope selection algorithm as previously described [23].

Briefly, potential HLA class I epitopes with high predicted binding affinity, high allele frequency, and high potential expression were selected. When possible, predicted neoepitopes for all patient HLA class I molecules were selected. Furthermore, peptides that potentially bind to multiple HLA class I molecules of the patient were preferred. In addition, possible HLA class II epitopes with a length of about 17 amino acids with high allele frequency and expected high expression were selected. These long neoepitopes were not predicted. Expression of respective proteins in breast cancer samples was manually checked in the Human Protein Atlas database (https://www.proteinatlas.org (accessed on 6 November 2014)) and integrated into the peptide selection process.

### 2.3. Vaccine Formulation and Administration

Peptides were synthesized by solid-phase peptide synthesis (SPPS) and purified to at least 95% (synthesized by EMC microcollections GmbH). Lyophilized peptides (HCl salt) were dissolved in water (Aqua injectibilia; BBraun, Melsungen, Germany) + 10% (or 33% for only patient 4) dimethylsulfoxide (DMSO). Peptides were mixed and sterile-filtered through a PTFE-membrane filter (Millex-LG sterile filter). The final concentration of the multipeptide solution was 0.8 mg/mL per peptide. The resulting peptide vaccine was controlled for identity and purity of contained peptides as well as sterility and absence of endotoxins followed by QC/QA release. Because synthetic peptides have demonstrated excellent feasibility and safety in earlier clinical trials, the safety profile was not tested before immunization.

Per vaccination, 0.5 mL of peptide solution was injected intracutaneously into the left or right lower abdomen followed by subcutaneous injection of 83 µg sargramostim (60% of the recommended average standard dose). After the 8th vaccination, sargramostim injection was followed by superficial application of imiquimod in the same area. Patient 3 did not receive imiquimod. The patients were vaccinated four times in the priming phase (one month) of the vaccination process with subsequent boosting vaccinations every four to six weeks.

### 2.4. Immune Monitoring

Immune monitoring was performed for detection of vaccine-induced T-cell responses. Peripheral blood mononuclear cells (PBMCs) were isolated from whole blood using Biocoll Separation Solution (Biochrom, Cambridge, UK). After density gradient centrifugation, PBMCs were washed and cryopreserved in freezing medium containing 10% DMSO (VWR, Darmstadt, Germany) until further usage. After thawing, PBMCs were cultivated for 12 h in TexMACS medium (Miltenyi Biotec, Bergisch-Gladbach, Germany) containing 3 µg/mL DNAse I (Sigma-Aldrich, St. Louis, MI, USA). After pre-incubation, cells were washed and re-sowed in TexMACS medium containing 1% penicillin-streptomycin (Sigma-Aldrich). Peptides were added at a concentration of 1 µg/mL for MHC class I peptides and 5 µg/mL for MHC class II peptides. Cells were cultivated in the presence of peptides for 12 days. After the first 24 h of cultivation, 10 U/mL IL-2 (Miltenyi Biotec, Bergisch-Gladbach, Germany) and 10 ng/mL IL-7 (Miltenyi Biotec) were added. Medium was changed every 2–3 days. After 12 days of cultivation, expanded cells were restimulated with corresponding peptides at the same concentration and additionally incubated for 14 h in the presence of Golgi inhibitors (Golgi Plug; BD biosciences, Franklin Lakes, NJ, USA; concentration: 1 µL/mL).

The readout was flow cytometric analysis after intracellular cytokine staining (ICS) as previously described [25]. Briefly, after cultivation, cells were washed and stained extra- and intracellularly using fluorochrome-conjugated antibodies titrated to their optimal concentrations. Finally, cells were measured on a Novocyte 3005R cytometer (Agilent).

Data were analyzed using FlowJo version 10.5.3 (FlowJo LLC, Ashland, AZ, USA). Briefly, CD4+ and CD8+ T-cells were gated within viable CD3+ lymphocytes and analyzed separately for each functional marker (CD154, IFN-γ, TNF, and IL-2). CD154 data were not available for patients 2 and 3 at time points V1 and V7. Peptide-specific responses were evaluated using the stimulation index (SI). The stimulation index is the calculated ratio of polyfunctional activated CD4+ or CD8+ T-cells (positive for at least 2 markers of CD154, IFN-γ, TNF, and /or IL-2) in the peptide-stimulated sample to the negative control sample (NC). Cells stimulated with 2 µg staphylococcal enterotoxin B (SEB; Sigma-Aldrich) served as positive control (PC). Neoantigen-specific T-cells were defined as being present if SI was ≥2. Additionally, a minimum frequency of 0.1% of reactive T-cells positive for at least one activation marker including CD154, IFN-γ, TNF, and/or IL-2 had to be reached among a minimum of 10,000 measured CD4+ or CD8+ events. We distinguished between solely CD4 responses, solely CD8 responses, as well as combined CD4 and CD8 responses. A detailed gating strategy can be found in Supplementary Figure S1.

## 3. Results

### 3.1. Demographic and Clinical Characteristics of the Patient Cohort

Four patients were included in this voluntary treatment attempt between November 2015 and August 2022.

Patient 1 was initially diagnosed with HER2-negative ER/PR negative (triple negative) high-grade (G3) invasive ductal carcinoma (pT1c pN0 cM0). Sequencing revealed a tumor mutational burden (TMB) of 4.4 mutations/megabase. Patient 2 was diagnosed with ER/PR positive/HER2 negative intermediate grade (G2) invasive ductal carcinoma of the left breast (pT4b pN3a cM0). The TMB was 1.8 mutations/megabase. Patient 3 was initially diagnosed with ER/PR positive/HER2 negative low-grade (G1) tubular mammary carcinoma of the right breast (pT1c cN0 cM0). The TMB was 0.3 mutations/megabase. Patient 4 was diagnosed with ER/PR negative/HER2 positive high-grade (G3) invasive ductal carcinoma of the right breast measuring 45 mm, without lymph node involvement and without distant metastasis (pT2, pN0, cM0). The TMB was 3.2 mutations/megabase.

**Figure 1:**
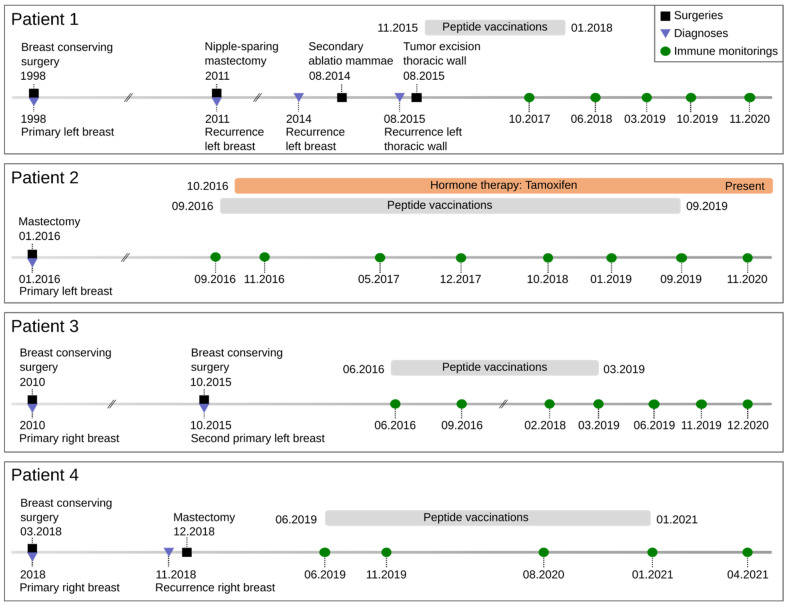
Timeline of clinical treatments. Onset of tumor treatment until end of peptide vaccinations. Blue triangles represent primary diagnosis, histology, staging, and tumor profile. Black squares indicate surgery. Gray bars define the course of peptide vaccination. Green circles exhibit the time points for immune monitoring during vaccinations. Orange bar shows the duration of systemic therapy.

The median age was 53 years at the start of treatment (range from 47 to 60). More patient information can be found in Figure 1. At the time point of submission, none of the patients showed evidence of tumor recurrence.

### 3.2. Vaccines

In total, 37 different peptides were synthesized, formulated, and vaccinated after QC/QA release. A detailed list of peptides can be found in Supplementary Table S1. The design and production of each individual vaccine was finished within 10 weeks after receipt of tumor tissue. Patients received between 17 and 40 vaccinations.

### 3.3. Safety

Adverse events (AEs) were mostly mild to moderate (grades 1 to 2). The most common AEs were local skin reactions such as redness, itching, and ulcer formation (Supplementary Figure S2).

Severe AEs (grade 3) were only observed in patient 1. After the 21st vaccination, the patient displayed strong local skin reactions and a weak systemic allergic reaction without requiring further medical support. None of the patients discontinued the treatment attempt because of an AE and no grade 4 AE was observed.

### 3.4. Immunogenicity

The presence of pre-existing and vaccine-induced CD4+ and CD8+ T-cell responses against the predicted neoantigens was determined by intracellular cytokine staining after in vitro expansion. Neoantigen-specific T-cells were evaluated at baseline (before the first vaccination, except patient 1) and during vaccination. Altogether, 37 different peptides were vaccinated (8–11 peptides/patient), thereof 26 short HLA class I peptides (abbreviated p*x*) and 11 long HLA class II peptides (abbreviated P_*x*) (4–10 and 1–4 peptides/patient, respectively).

**Figure 2:**
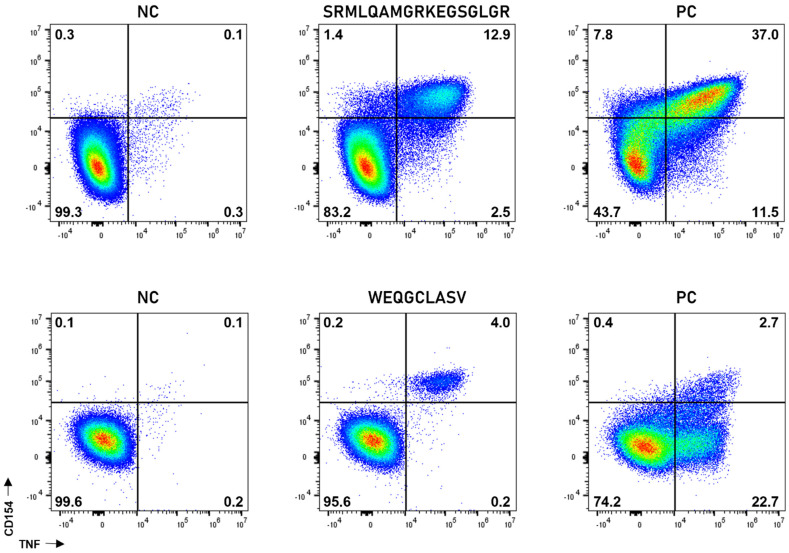
Peptide-specific CD4+ and CD8+ T-cell responses are robust and polyfunctional. Representative immune monitoring examples from patient 4. Vaccine-induced peptide-specific CD4+ T-cells ((**upper row**): P_36) and CD8+ T-cells ((**lower row**): p30) are polyfunctional (x-axis: TNF; y-axis: CD154). Mock restimulated sample (negative control, NC: (**left**)), peptide-restimulated cells (**middle**), and SEB-stimulated sample (positive control, PC: (**right**)) are shown. Numbers indicate frequency within all CD4+ or CD8+ T-cells, respectively.

**Figure 3:**
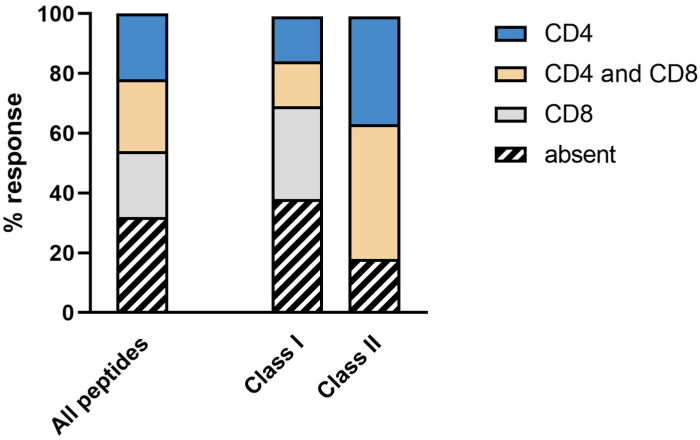
Immunogenicity of vaccinated peptides. Percentages of durable vaccine-induced immune responses. In total, 37 different peptides were vaccinated (26 HLA class I peptides and 11 HLA class II peptides). We distinguished between immune responses solely mediated by CD4+ (blue), immune responses solely mediated by CD8+ (gray), immune responses mediated by both CD4+ and CD8+ (sand), and no response (black shaded). Immune responses were defined as being durable if they were detected at two time points.

Generally, neoantigen-derived peptide vaccines were able to induce robust CD4+ and CD8+ T-cell responses. Examples of peptide-specific CD4+ and CD8+ T-cell responses are shown in Figure 2. The vaccine induced (i) solely CD4+ T-cell response against 7 of 37 peptides (19%), (ii) solely CD8+ T-cell response against 5 of 37 peptides (14%), and (iii) combined CD4+ and CD8+ T-cell response against 11 of 37 vaccinated peptides (30%). A total of 37% of all vaccinated peptides (14 of 37 neoantigens) did not induce immune responses (Figure 3). Immune responses were defined as being durable if they were detected at least at two time points.

**Figure 4:**
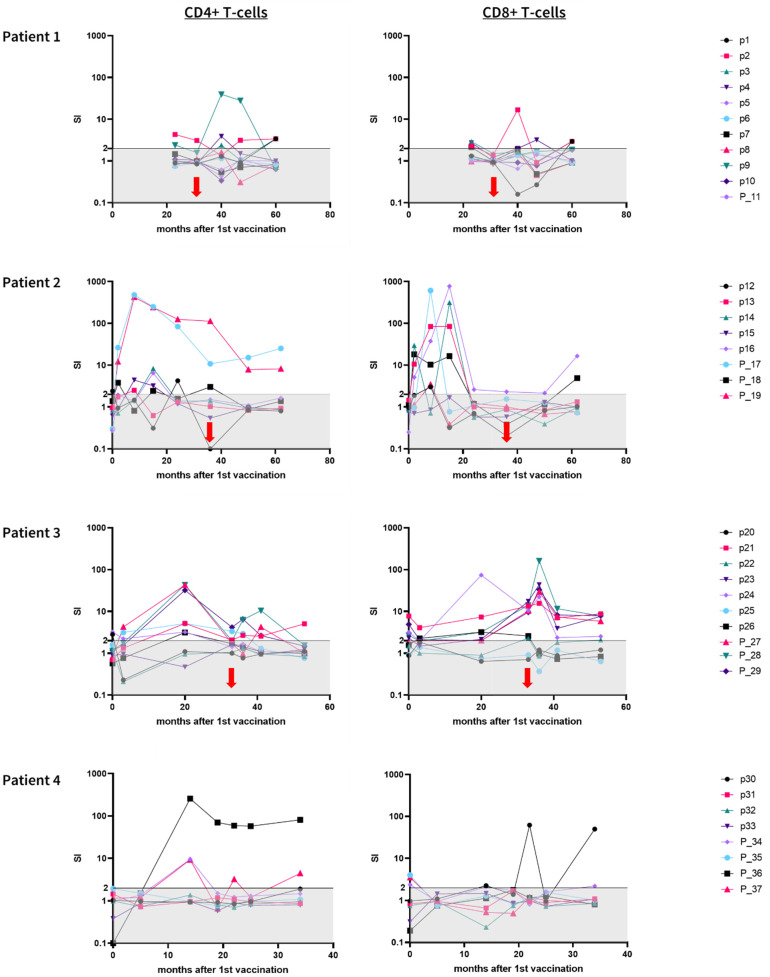
Stimulation index (SI) of vaccine-induced immune responses among individual patients over the course of vaccination. We distinguished between CD4+ (**left column**) and CD8+ (**right column**) immune responses. Neoantigen-specific T-cells were defined as being present if SI was ≥2 (gray area). p = short HLA class I peptide; P_ = long HLA class II peptide. Red arrow: date of last vaccination.

All three patients (the pre time point was not available for patient 1) showed weak pre-existing immune responses against altogether 10 of 37 (27%) peptides (Figure 4).

The vaccine for *patient 1* contained eleven peptides (ten short peptides and one long peptide). Pre-treatment immune monitoring is not available for this patient. The first instance of immune monitoring was performed at the 22nd vaccination (23 months after the 1st vaccination). We detected a robust and durable CD4+ T-cell response against one peptide (p9), a CD8+ T-cell response against one peptide (p4), and combined CD4+ and CD8+ T-cell responses against two peptides (p2 and p3).

The vaccine for *patient 2* contained eight peptides (five short peptides and three long peptides). Patient 2 showed a pre-existing CD4+ immune response against one short peptide (p12). On-treatment immune monitoring showed CD4+ T-cell responses against four peptides (p12, p15, P_17, and P_19), CD8+ T-cell responses against three peptides (p13, p14, and p16), and a combined CD4+ and CD8+ T-cell response against one peptide (P_18). Twenty-six months after the last vaccination, two CD4+ T-cell responses (P_17, P_19) and two CD8+ T-cell responses (p16, P_18) were still detectable.

The vaccine for *patient 3* contained ten peptides (seven short peptides and three long peptides). Pre-existing T-cell responses were detected against four short peptides and two long peptides. Immune monitoring performed during therapy revealed a CD4+ T-cell response against one peptide (p25), CD8+ T-cell responses against three peptides (p22, p23, and p26), and combined CD4+ and CD8+ T-cell responses against five peptides (p21, p24, P_27, P_28, and P_29). Twenty months after the last vaccination, six CD8+ T-cell responses (p22, p23, p24, P_27, P_28, and P_29) and one combined CD4+ and CD8+ T-cell response (p21) were still detectable.

The vaccine for *patient 4* contained eight peptides (four short peptides and four long peptides). Pre-treatment immune monitoring showed CD8+ T-cell responses against three long peptides. During therapy, immune monitoring was performed between 5 and 34 months after the 1st vaccination. A CD4+ T-cell response was detected against two peptides (P_36 and P_37), a CD8+ T-cell response was detected against one peptide (p30), and a combined CD4+ and CD8+ T-cell response was detected against one peptide (P_34). The patient did not finish treatment until the last follow-up.

## 4. Discussion

Our increasing understanding of interactions between tumors and the immune system has established immunotherapy as the fourth pillar of cancer treatment in recent years. However, most cancer patients still do not profit from immunotherapeutic approaches such as checkpoint blockade. Thus, personalized vaccination against tumor-specific neoantigens is a rapidly growing field. The aim of our study was to show the safety and feasibility of the production and application of a fully individualized cancer peptide vaccine in real-life conditions. Four female breast cancer patients under remission received neoantigen-derived peptide vaccine therapy as recurrence prophylaxis under compassionate use. Personalized peptide vaccines were designed based on DNA and RNA sequencing data, the HLA type of the patient, and predicted peptide binding affinities. Both HLA class I and II peptides were selected. Immune monitoring was implemented periodically to confirm vaccination-induced CD4+ and CD8+ T-cell responses.

Recent neoantigen vaccination trials were mainly performed in high TMB tumors, such as melanoma, to ensure the identification of a sufficient number of target neoantigens [26,27]. However, even though breast carcinoma has, in general, much lower TMB values compared with melanoma, our approach was able to identify an adequate number of neoantigens. Currently, standard-of-care breast cancer treatment mainly involves surgery and hormone and growth factor receptor targeting (HER2 and ER/PR). Although there are already many therapy options available for breast cancer treatment, neoantigen-derived vaccines might be promising for those subtypes where therapeutic options are scarce [15]. Furthermore, our results suggest that neoantigen-derived vaccines can potentially be applied in cancer entities with even lower TMB values than breast carcinoma.

In former trials, identified (neo) antigens have been administered in different formats including peptides [27], DNA [28] and RNA [26], or preloaded in vitro on dendritic cells [29]. Here we decided to use a peptide-based vaccine as, earlier, we have successfully generated robust anti-tumor T-cell responses in a pancreatic cancer patient [21]. Furthermore, synthetic peptides have the advantage of being well-defined and relatively inexpensive to synthesize.

During therapy, all four patients exhibited durable and strong vaccine-induced CD4+ and CD8+ T-cell responses against several peptides including relevant targeted driver mutations. These T-cell responses were polyfunctional, meaning that at least two of the four measured activation markers were expressed simultaneously after peptide stimulation. Altogether, the majority of vaccinated peptides did induce an immune response (63%) and the observed responses were mainly CD4+ driven. Interestingly, similar results were observed by Ott et al. in melanoma patients undergoing peptide vaccination [30]. Notably, weak T-cell responses against single peptides were detectable in all patients analyzed before treatment. These pre-existing responses are the result of sporadic T-cell priming before therapy against autologous tumor-specific antigens, clearly demonstrating the appropriateness of our target selection approach.

We observed no serious adverse events during vaccination in these patients. This was in concordance with previous trials using peptide-based vaccines together with sargramostim [31,32]. Besides common “flu-like” symptoms or local reactions, no patient reported severe negative effects. No attempt had to be stopped due to severe AEs. Furthermore, we have shown that it is technically feasible to produce a potent neoantigen-derived peptide vaccine. Production and formulation of each individual vaccine was finished about 10 weeks after enrollment. T-cell responses were induced or enhanced without any additional treatment, such as anti-PD-1 therapy. Importantly, some peptides elicited long-lasting T-cell responses that were detectable even months after the last vaccination. Notably, all patients remained in remission, which indicates the possible potential of neoantigen-derived peptide vaccines to be used for long-term prophylaxis. Currently, we are considering further analyses with larger patient cohorts to show the specific effect of the neoantigen vaccine on protection against the recurrence of disease [33,34].

Our study has several important limitations. Firstly, the small sample size. Secondly, due to minor individual adverse events, two adjuvants (sargramostim and imiquimod) were applied differently among patients. Thirdly, patients received different numbers of vaccinations.

Fourthly, we cannot specifically estimate long-term clinical effects as the observation period is not long enough. Although we were able to detect neoantigen-specific T-cells several months after the last vaccination, the follow-up time in our study was too short to encompass all likely cases of remission.

Finally, the small number of patients does not allow for statistical conclusions. Our main conclusion is that the production of a personalized peptide vaccine is feasible. We have evidence that our vaccine approach is safe and that it generated durable immune responses in the patients that were enrolled.

There are a number of additional research questions that have emerged and may warrant further studies in larger cohorts, such as the role of adjuvants, optimization of prediction algorithms, optimal ratio/amount of HLA class I vs. II peptides, and the ideal vaccination schedule. If more promising data can be obtained, peptide vaccines have the potential to be utilized not only in the complementary adjuvant setting but also as an interventional treatment in newly diagnosed patients.

## Data Availability

The data presented in this study are available on reasonable request from the corresponding author. The data are not publicly available because they contain information that could compromise the privacy of the research participants and third-party restrictions.

## Supplementary Materials

The following supporting information can be downloaded at: https://www.mdpi.com/article/10.3390/vaccines10111882/s1, Figure S1: Gating strategy, Figure S2: Local skin reactions and Table S1: List of vaccinated peptides and results immune monitoring.
