# Supplementary material for: Adjuvant Treatment for Breast Cancer Patients Using Individualized Neoantigen Peptide Vaccination—A Retrospective Observation
Source: Vaccines (Basel). 2022 Nov 8;10(11):1882. doi: 10.3390/vaccines10111882 (PMC9698403; doi:10.3390/vaccines10111882)
Supplement: Supplementary file 1 [file vaccines-10-01882-s001.zip › vaccines-1961022-supplementary.pdf]

Supplementary Material for:

Adjuvant treatment for breast cancer patients using individualized neoantigen peptide vaccination – a retrospective observation

Author List and Affiliations

Henning Zelba[1,*,#], Alex McQueeney[1,#], Armin Rabsteyn[1], Oliver Bartsch[1], Christina Kyzirakos[1], Simone Kayser[1], Johannes Harter[2], Pauline Latzer[1], Dirk Hadaschik[3], Florian Battke[2], Andreas Hartkopf[4], Saskia Biskup[1,2]

1. Zentrum fuer Humangenetik, Tuebingen, Germany
2. CeGaT GmbH, Tuebingen, Germany
3. Cecava GmbH, Tuebingen, Germany
4. Department of Obstetrics and Gynaecology, University of Tuebingen, Tuebingen, Germany

* Correspondence: Henning.Zelba@humangenetik-tuebingen.de

\# Authors contributed equally

Figure S1: Gating strategy

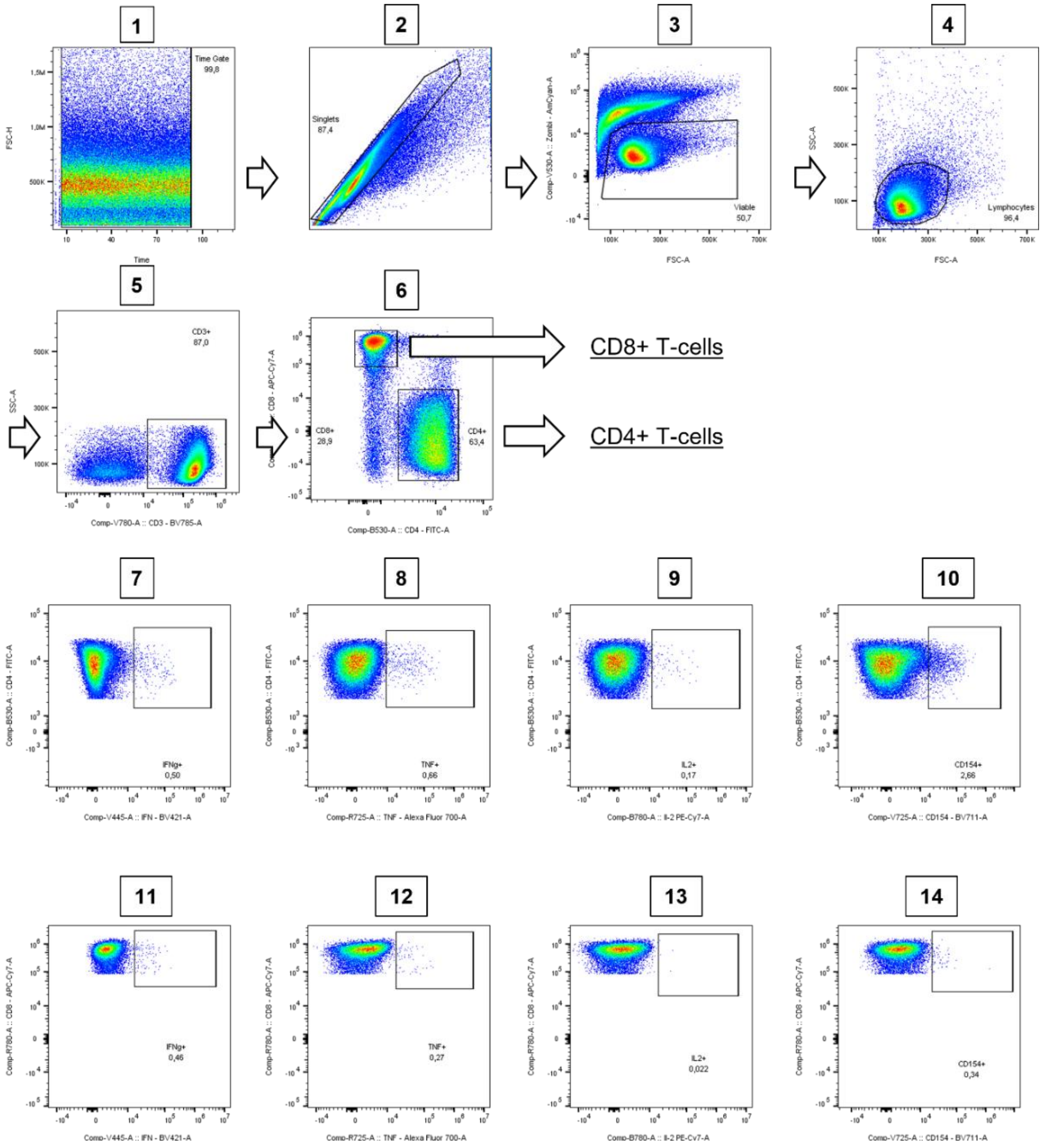

We included only cells that were constantly measured over time (1; Forward-scatter (FSC)-H versus Time). Herein, single (2; FSC-A versus FSC-H), viable (3; Zombie Aqua-negative cells), lymphocytes (4; FSC-A versus Side-scatter (SSC)-A) and CD3+ T-cells (5) were selected. CD3+ T-cells were further discriminated in CD4+ or CD8+ T-cells (6). Within both CD4+ (7-10) and CD8+ T-cells (11-14), we determined the production/expression of the functional markers IFN-γ (7, 11), TNF (8, 12), IL-2 (9, 13) and CD154 (10, 14).

Figure S2: Local skin reactions

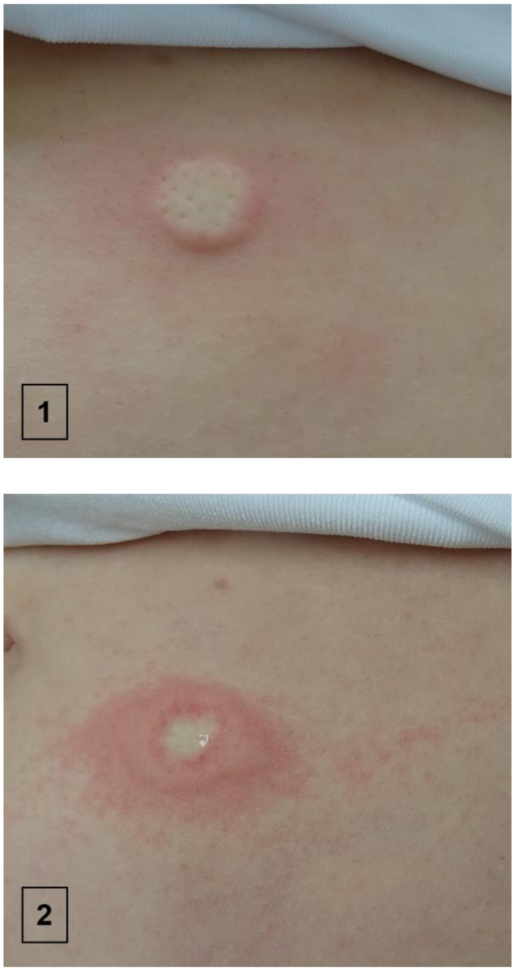

Injection site of *patient 4* (18th vaccination) immediately after application (1) and after 10 minutes (2). Per vaccination, 0.5 ml peptide solution were injected intracutaneously in the lower abdomen followed by subcutaneous injection of 83 µg Sargramostim.

Table S1: List of vaccinated peptides and results immune monitoring

Patient	PeptideID	AA seqeunce	Gene	HLA	VAF DNA	Preexisting		IMM1		IMM2		IMM3		IMM4		IMM5		IMM6		IMM7	
						CD4	CD8	CD4	CD8	CD4	CD8	CD4	CD8	CD4	CD8	CD4	CD8	CD4	CD8	CD4	CD8
1	p1	VLKQVHTDTGI	HIST1H2BN:NM_003520:c.C151A:p.P51T	HLA-A*02:01	0,32	n.a.	n.a.	0	0	0	0	0	0	0	0	1	1	n.a.	n.a.	n.a.	n.a.
1	p2	KYLFYPQFLW	PLA2G10:NM_003561:c.T471G:p.C157W	HLA-A*23:01	0,31	n.a.	n.a.	1	1	1	0	0	1	1	0	1	1	n.a.	n.a.	n.a.	n.a.
1	p3	ATLSRIPHI	CHD3:NM_005852:c.C5570A:p.P1857H	HLA-B*49:01, HLA-A*02:01	0,31	n.a.	n.a.	0	1	0	0	1	0	0	0	1	1	n.a.	n.a.	n.a.	n.a.
1	p4	TSRDIRIVF	KIAA0922:NM_015196:c.C2445G:p.S815R	HLA-B*15:01	0,31	n.a.	n.a.	0	1	0	0	1	1	0	1	0	0	n.a.	n.a.	n.a.	n.a.
1	p5	FANTLGKPEY	ERBB4:NM_001042599:c.G3607C:p.A1203P	HLA-B*15:01	0,24	n.a.	n.a.	0	0	0	0	0	0	0	0	0	0	n.a.	n.a.	n.a.	n.a.
1	p6	SLYFGNEEEL	TVP23A:NM_001079512:c.G43T:p.D15Y	HLA-A*02:01	0,18	n.a.	n.a.	0	0	0	0	0	0	0	0	0	0	n.a.	n.a.	n.a.	n.a.
1	p7	KYAELTVVREL	PCDH18:NM_001300828:c.T596C:p.I199T	HLA-A*02:01, HLA-A*23:01	0,16	n.a.	n.a.	0	1	0	0	0	0	0	0	0	0	n.a.	n.a.	n.a.	n.a.
1	p8	STLNEELGQV	ATP8B2:NM_020452:c.C1229G:p.T410S	HLA-A*02:01	0,13	n.a.	n.a.	0	0	0	0	0	0	0	0	0	0	n.a.	n.a.	n.a.	n.a.
1	p9	CETNRMDGAV	NCOA1:NM_147233:c.G2500A:p.D834N	HLA-B*49:01	0,13	n.a.	n.a.	1	0	0	0	1	0	1	0	0	0	n.a.	n.a.	n.a.	n.a.
1	p10	HELTVGNRLI	GABRP:NM_014211:c.G397C:p.V133L	HLA-B*49:01	0,20	n.a.	n.a.	0	0	0	0	0	0	0	0	0	0	n.a.	n.a.	n.a.	n.a.
1	P_11	MTEVVRRCERCSDSDG	TP53:NM_001126112.2:c.529_537del:p.P177_H179del	class II	0,22	n.a.	n.a.	0	0	0	0	0	0	0	0	0	0	n.a.	n.a.	n.a.	n.a.
2	p12	ATGGHLCQQPK	MUC5B:NM_002458:c.G4606A:p.A1536T	HLA-A*03:01	0,21	1	0	0	0	0	1	0	0	1	0	0	0	0	0	0	0
2	p13	VVMDLKKCR	CHD4:NM_001273:c.T3055C:p.C1019R	HLA-A*31:01	0,11	0	0	0	1	1	1	0	1	0	0	0	0	0	0	0	0
2	p14	FMKQMNDAR	PIK3CA:NM_006218:c.A3140G:p.H1047R	HLA-A*31:01	0,11	0	0	0	1	0	0	1	1	0	0	0	0	0	0	0	0
2	p15	LAWTAMGGF	LONP1:NM_001276480:c.C1739T:p.S580F	HLA-B*35:01	0,13	0	0	0	0	1	0	1	0	0	0	0	0	0	0	0	0
2	p16	FPKIVAFETV	ALAS1:NM_199166:c.G1123T:p.V375F	HLA-B*51:01	0,10	0	0	0	1	0	1	1	1	0	1	0	1	0	1	0	1
2	P_17	FMKQMNDARHGGWTTKM	PIK3CA:NM_006218:c.A3140G:p.H1047R	class II	0,11	0	0	1	0	1	1	1	0	1	0	1	0	1	0	1	0
2	P_18	LLQRSDPSFPKIVAFET	ALAS1:NM_000688.5:c.G1123T:p.V375F	class II	0,10	0	0	1	1	0	1	1	1	0	0	1	0	0	0	0	1
2	P_19	GPVLREEAKAGPGLQGG	ZXDB:NM_007157.3:c.[G364AA368C]:p.[E122KE123A]	class II	0,14	0	0	1	0	1	1	1	0	1	0	1	0	1	0	1	0

3 p20	NPRTGGERGFL	EXT1:NM_000127:c.C721A:p.H241N	HLA-B*07:02	0,43	1	0	0	0	0	0	0	0	0	0	0	0	0	0	n.a.	n.a.
3 p21	MSYQGLPSTQL	NOTCH1:NM_017617:c.G7115A:p.R2372Q	HLA-C*02:02, HLA-C*12:03	0,90	0	1	0	1	1	1	1	1	1	1	1	1	1	1	n.a.	n.a.
3 p22	RVEGNLRVEDL	TP53:NM_001126112:c.T613G:p.Y205D	HLA-A*02:01	0,95	0	0	0	0	0	0	0	1	0	0	0	0	0	1	n.a.	n.a.
3 p23	FPYLTGQMAM	VEPH1:NM_001167911:c.G875T:p.R292M	HLA-C*12:03, HLA-B*07:02, HLA-C*02:02	0,30	0	1	0	0	0	1	0	1	0	0	0	0	0	1	n.a.	n.a.
3 p24	ARFMYVTEL	CCDC63:NM_152591:c.C1013T:p.T338M	HLA-B*27:02	0,80	1	1	1	0	1	1	0	1	0	0	0	0	0	1	n.a.	n.a.
3 p25	MLMTVKQYEAA	ARFIP2:NM_012402:c.C662T:p.T221M	HLA-A*02:01	0,83	0	0	1	0	1	0	1	0	0	0	0	0	0	0	n.a.	n.a.
3 p26	TTYDLSSV	CARM1:NM_199141:c.G1549A:p.A517T	HLA-C*02:02, HLA-A*02:01, HLA-C*12:03	0,81	0	0	0	1	1	1	0	1	0	0	0	0	0	0	n.a.	n.a.
3 P_27	VEGNLRVEDLDDRNTFR	TP53:NM_001126112:c.T613G:p.Y205D	class II	0,95	0	0	1	0	1	1	0	1	0	1	1	1	0	1	n.a.	n.a.
3 P_28	NNEISLRDIEVYGFDYD	NT5DC2:NM_022908.2:c.199G>A:p.V67I	class II	0,82	0	1	0	0	1	1	0	1	1	1	1	1	0	1	n.a.	n.a.
3 P_29	KLPASRSICNLFGSGSL	KIF1A:NM_004321.6:c.3913C>T:p.R1305C	class II	0,81	0	1	0	1	1	1	1	1	1	1	1	1	0	1	n.a.	n.a.
4 p30	WEQGCLASV	NFKBIA:NM_020529.2:c.T456TG:p.C152W	HLA-A*02:01, HLA-B*40:01	0,72	0	0	0	0	0	1	0	0	0	0	0	0	1	1	n.a.	n.a.
4 p31	HAAEPARDCV	TFE3:NM_006521.5:c.G31T:p.G11C	HLA-A*02:01	0,16	0	0	0	0	0	0	0	0	0	0	0	0	0	0	n.a.	n.a.
4 p32	AMGRKEGSGL	RBM10:NM_005676.4:c.T2611TA:p.W871R	HLA-A*02:01	0,19	0	0	0	0	0	0	0	0	0	0	0	0	0	0	n.a.	n.a.
4 p33	VLQILRVSL	MSH5:NM_025259.5:c.750delT:p.F250Lfs*16	HLA-A*02:01, HLA-B*40:01	0,32	0	0	0	0	0	0	0	0	0	0	0	0	0	0	n.a.	n.a.
4 P_34	GNTPLHLAWEQGCLASVGVL	NFKBIA:NM_020529.2:c.T456G:p.C152W	class II	0,72	0	1	0	0	1	1	0	0	0	0	0	0	0	1	n.a.	n.a.
4 P_35	HAAEPARDCVEASAEGP	TFE3:NM_006521.5:c.G31T:p.G11C	class II	0,16	0	1	0	0	0	0	0	0	0	0	0	0	0	0	n.a.	n.a.
4 P_36	SRMLQAMGRKEGSGLGR	RBM10:NM_005676.4:c.T2611A:p.W871R	class II	0,19	0	0	0	0	1	0	1	0	1	0	1	0	1	0	n.a.	n.a.
4 P_37	SQHMTEVVRHCPHHER	TP53:NM_000546.5:c.G524A:p.R175H	class II	0,81	0	1	0	0	1	0	0	0	1	0	0	0	1	0	n.a.	n.a.

AA: amino acid
HLA: HLA which was predicted to bind the peptide
NAF: Novel allele frequency, frequency with which the mutated allele was occurring in the tumor sequencing (1 is 100%). The observed frequencies are influenced by the tumor content of the analyzed sample and hence do not correlate directly to the mutation frequency in the tumor
IMM: Immune monitoring
n.a.: not available
1: Response detected
0: no response detected
